# Structural Determinants of the Stability of Enzyme‐Responsive Polyion Complex Nanoparticles Targeting *Pseudomonas aeruginosa*’s Elastase

**DOI:** 10.1002/cnma.201800054

**Published:** 2018-04-23

**Authors:** Ignacio Insua, Marion Petit, Lewis D. Blackman, Robert Keogh, Anaïs Pitto‐Barry, Rachel K. O'Reilly, Anna F. A. Peacock, Anne Marie Krachler, Francisco Fernandez‐Trillo

**Affiliations:** ^1^ School of Chemistry University of Birmingham Edgbaston B15 2TT UK; ^2^ Institute of Microbiology and Infection – School of Biosciences University of Birmingham Edgbaston B15 2TT UK; ^3^ Department of Chemistry University of Warwick Coventry CV4 7AL UK; ^4^ Current address: Department of Microbiology and Molecular Genetics University of Texas Health Science Center Houston (TX) USA; ^5^ Current address: School of Chemistry and Biosciences University of Bradford Bradford BD7 1DP

**Keywords:** Nanoparticles, Enzymes, Polyelectrolytes, Self-assembly, Drug delivery

## Abstract

Here, we report how the stability of polyion complex (PIC) particles containing *Pseudomonas aeruginosa*’s elastase (LasB) degradable peptides and antimicrobial poly(ethylene imine) is significantly improved by careful design of the peptide component. Three LasB‐degradable peptides are reported herein, all of them carrying the LasB‐degradable sequence −GLA− and for which the number of anionic amino acids and cysteine units per peptide were systematically varied. Our results suggest that while net charge and potential to cross‐link via disulfide bond formation do not have a predictable effect on the ability of LasB to degrade these peptides, a significant effect of these two parameters on particle preparation and stability is observed. A range of techniques has been used to characterize these new materials and demonstrates that increasing the charge and cross‐linking potential of the peptides results in PIC particles with better stability in physiological conditions and upon storage. These results highlight the importance of molecular design for the preparation of PIC particles and should underpin the future development of these materials for responsive drug delivery.

## Introduction

Polyion complex (PIC) (nano)particles, also known as polyelectrolyte complexes (PECs)^1^ or interpolyelectrolyte complexes (IPECs),^2^ are soft colloids obtained from the self‐assembly of oppositely charged polyelectrolytes in solution.^3^ These nanomaterials are attractive vehicles for the delivery of charged drugs such as antineoplastics,^4^, ^5^, ^6^, ^7^ antimicrobials^8^, ^9^, ^10^, ^11^, ^12^, ^13^, ^14^ and nucleic acids,^15^ which can be complexed with oppositely charged polymers to form PIC (nano)particles that reduce the toxicity and/or control the activity of these drugs. Given the key role of enzymes in many diseases and their remarkable specificity,^16^ PIC (nano)particles prepared from polyelectrolytes that degrade in the presence of these enzymes have a great potential for biomedical applications. This approach is particularly interesting to tackle infections, since many pathogens secrete enzymes (e. g. proteases) to overcome the host defenses.^17^ By incorporating enzyme‐responsive components into the PIC (nano)particle, the release of the antimicrobial can be localized to the surroundings of the pathogenic organism, leading to improved therapeutic profiles.

**Figure 1:**
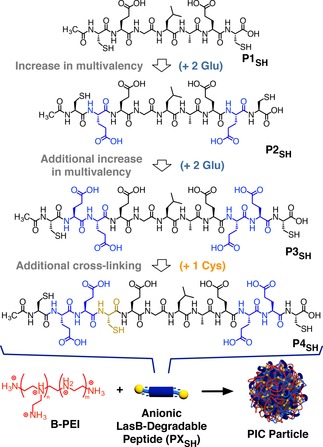
Structures of the LasB‐degradable peptides evaluated in this work and their self‐assembly with branched poly(ethylene imine) (B‐PEI) to form antimicrobial PIC particles.

With these principles in mind, we have recently reported the preparation of enzyme‐responsive PIC nanoparticles for the targeted delivery of antimicrobial branched poly(ethylene imine) (B‐PEI), which was released upon exposure to *Pseudomonas aeruginosa*’s elastase LasB.^18^ To this end, anionic peptide **P1_SH_** (Figure 1) was designed, that contained the amino acid sequence glycine‐leucine‐alanine (−GLA−), which is hydrolyzed by LasB. This sequence was inserted in between two glutamic acids (E), responsible for the electrostatic interaction with B‐PEI, and two cysteines (C), to cross‐link via disulfide formation once the nanoparticle is formed. The reported PIC nanoparticles showed excellent potential for the targeted delivery of B‐PEI to *P. aeruginosa*, being specifically degraded by the bacterial elastase and displaying a LasB‐specific activity against *P. aeruginosa*. However, the overall antimicrobial activity of the particles was low, with only 20% of the activity of free B‐PEI recovered in the presence of *P. aeruginosa*. Due to the low multivalency of peptide **P1_SH_**, only a small range of formulations resulted in the formation of stable PIC nanoparticles, thus compromising optimization of the delivery system.

In this article, we present our efforts to improve the stability of these LasB‐responsive PIC particles by optimizing the structure of the peptide component. Three new LasB‐responsive peptides were prepared with increasing number of anionic residues and cross‐linking groups (Figure 1, **P2_SH_**‐**P4_SH_**). The hydrolysis of peptides **P2_SH_‐P4_SH_** by LasB was evaluated and compared to that caused by a model human elastase. While the extent and specificity of hydrolysis was influenced by their amino acid sequence, no clear correlation between multivalency and susceptibility to LasB was observed. When mixed with the antimicrobial B‐PEI, all these peptides were able to give PIC particles across a wide range of formulations. Representative PIC particles were characterized using a range of light scattering techniques, suggesting that no significant structural differences could be observed between nanoparticles. Finally, the stability of these nanoparticles under simulated physiological conditions or under storage was also evaluated. Overall, particles prepared with the new peptides **P2_SH_**‐**P4_SH_** showed better stability than those obtained from **P1_SH_**, with increasing stability in simulated physiological conditions as the multivalency of the peptides was increased.

## Results and Discussion

### Peptide Design and Synthesis

Based on the structure of LasB‐responsive peptide **P1_SH_** (Ac‐C‐E‐GLA‐E‐C‐OH), previously reported by our group,^18^ two modifications were proposed to enhance the saline stability of the resulting PIC nanoparticles: i) Additional glutamic acids were introduced to increase the number of anionic residues (Figure 1, **P2_SH_** and **P3_SH_**), potentially leading to a stronger affinity for antimicrobial B‐PEI; and ii) the incorporation of a third cysteine (Figure 1, **P4_SH_**), which is expected to increase the cross‐linking density via disulfide formation once the PIC nanoparticles are formed. Both charge density and cross‐linking density have been reported as factors contributing to the stability of this type of nanoparticles.^3^ Overall, the peptides prepared included either two additional glutamic acids (**P2_SH_**), four additional glutamic acids (**P3_SH_**), or four additional glutamic acids and an extra cysteine (**P4_SH_**) compared to the parent peptide **P1_SH_**. All peptides were synthesized by solid‐phase chemistry in good to excellent yields and high purity without any chromatographic purification (see SI: Figures S1–S3† for experimental details and characterization).

### Enzymatic Degradation of Peptides

**Figure 2:**
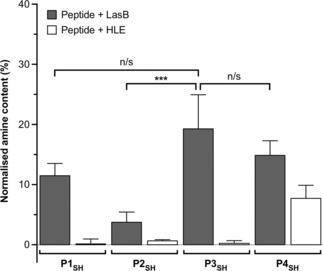
Relative amine content in samples of LasB responsive anionic peptides evaluated in this work. Relative amine content was calculated from fluorescamine conjugates formed following incubation with enzymes for 4 hours and normalized to the fluorescence observed with a model degradation peptide (**P5**, H_2_N‐LA‐E‐OH) (Figure S4). *n*=3, mean values± standard deviation. One‐way ANOVA, followed by Tukey's test (CI=95%,) was used to test for significance. Statistical significance was determined ‘n/s’=not significant, *** p<0.001.

Next, the degradation of peptides **P1_SH_**–**P4_SH_** by *P. aeruginosa*’s elastase (LasB) was evaluated. This experiment involved quantification of the number of primary amines formed as a result of peptide hydrolysis, using the fluorescent reporter fluorescamine.^19^ *P. aeruginosa*’s LasB hydrolyses peptides containing the −GLA− sequence between the glycine and leucine residues.^20^ In these experiments, the fluorescent intensity of all fluorescamine adducts (Figure S4) was compared to that observed in the presence of H_2_N‐LAE‐OH (**P5**). **P5**’s sequence should be formed following LasB‐mediated hydrolysis of the −GLAE− sequence, present in all the peptides reported. Thus, 100% hydrolysis was assigned to the fluorescent intensity of the fluorescamine adduct of **P5** and all other intensities reported as a percentage of this one (Figure 2). The degradation of peptides **P1_SH_**–**P4_SH_** in the presence of Human Leukocyte Elastase (HLE), a protease released by white blood cells during *P. aeruginosa* infections,^21^ was also evaluated. This comparison allowed us to assess if these peptides were selectively hydrolyzed by the bacterial elastase over a relevant human enzyme.

All peptides were hydrolyzed by LasB, in agreement with our previous work that demonstrated that the addition of extra amino acids around the −GLA− tripeptide did not seriously compromise the activity of LasB.^18^ We anticipated that increasing the number of glutamic acids could have a detrimental effect on the activity of LasB, due to the potential sequestration of Ca^2+^ by carboxylic acids. Ca^2+^ is a LasB cofactor and polycarboxylated compounds, in our case ethylenediaminetetraacetic acid (EDTA), are commonly used to quench the activity of the enzyme. However, no obvious trend was observed for our peptides. **P1_SH_**, **P3_SH_** and **P4_SH_** displayed the highest susceptibility to LasB hydrolysis, with no statistical difference between them (Figure 2). Interestingly, **P2_SH_** was the least active of the peptides tested, despite having an intermediate number of glutamic acid residues. The potential of these peptides to adopt different conformations in solution that could explain this difference in activity was evaluated using circular dichroism (CD). However, CD suggested that all peptides had a random/extended conformation in solution without any significant difference across the collection (Figure S5A).^22^ Finally, introducing an additional cysteine did not have a big effect on the activity of LasB, with the number of amines obtained from the hydrolysis of **P4_SH_** only 5% smaller than those obtained from **P3_SH_**. However, this extra cysteine had a significant effect on the specificity of the peptide with **P4_SH_** being the only peptide significantly degraded by HLE (*ca*. 50% of the hydrolysis observed with LasB, Figure 2). This loss of specificity is in agreement with the ability of HLE to cleave at cysteine residues,^23^,^24^ while LasB's activity is moderately inhibited by thiols and cysteine residues.^25^, ^26^, ^27^

### Self‐assembly and Characterization of PIC Particles

**Figure 3:**
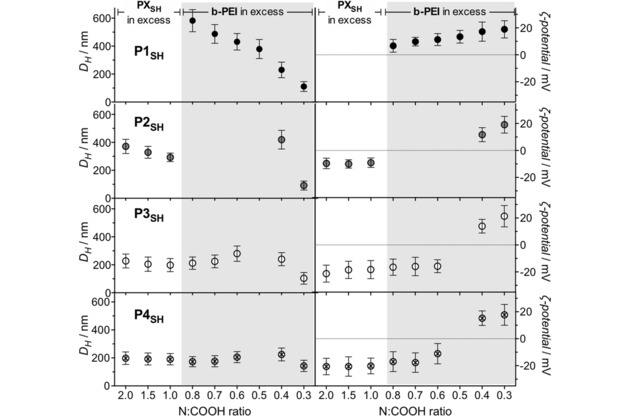
Hydrodynamic diameter (*D_H_*, left) and ζ‐potential (right) of PIC nanoparticles prepared from peptides **P1_SH_**–**P4_SH_** at different N : COOH ratios. Each value represents the mean size and charge of the only population fitted by the software for each sample ± its standard deviation. Empty spaces indicate formulations that did not form PIC particles. Further details can be found in Table S1. Results obtained directly after the assembly of the nanoparticles without prior filtration. Data for **P1_SH_** reproduced from I. Insua, E. Liamas, Z. Zhang, A. F. Peacock, A. M. Krachler, F. Fernandez‐Trillo, *Polym Chem* **2016**, *7*, 2684–2690 – Published by The Royal Society of Chemistry.

Having established that all peptides were hydrolyzed by LasB, we then explored their suitability for the preparation of B‐PEI containing PIC nanoparticles. These nanoparticles were prepared by mixing peptides **P1_SH_**–**P4_SH_** with B‐PEI in aqueous medium at physiological pH as previously reported for **P1_SH_**.^18^ The colloidal stability of PIC particles is highly dependent on their charge ratio (i. e. the relative number of positive and negative charges mixed in the formulation). Therefore, several formulations were explored for each peptide, where the relative number of cationic amines in B‐PEI *versus* anionic carboxylic acids in peptides **P1_SH_**–**P4_SH_** (N : COOH ratio) was systematically varied (Figure 3, Table S1). From their ζ‐potentials, formulations of these LasB‐responsive peptides could be split into 3 groups: a) *Formulations resulting in the formation of cationic nanoparticles*: While this was the case for all of the formulations made from **P1_SH_** (Figure 3, top), only those formulations with the smallest amounts of **P2_SH_**–**P4_SH_** peptides resulted in particles with a positively charged corona (Figure 3, 0.4 and 0.3 N : COOH ratios). b) *Formulations that yielded negatively charged nanoparticles*: We had previously reported that **P1_SH_** was unable to form negatively charged particles (Figure 3, top),^18^ possibly because of its small size and multivalency. We were therefore very pleased to see that increasing the multivalency for **P2_SH_**–**P4_SH_**, resulted in particles with a negatively charged corona for a broad range of formulations (Figure 3). This was particularly the case for **P3_SH_** and **P4_SH_**, the two peptides with the highest multivalency of the ones reported, which yielded negatively charged particles for formulations with N : COOH ratios ≥0.6. c) *A region where no PIC nanoparticles formed* in agreement with the mechanism of nucleation for this type of supramolecular aggregates.^2^,^3^,^28^ When mixed, polyelectrolytes will interact to form a neutral core made of a stoichiometric mixture of oppositely charged polyelectrolytes, surrounded by a corona of whichever polyion is present in excess. Neutral complexes that lack a charged stabilizing corona will not be colloidally stable and flocculation of particles will occur. While this phenomenon was observed at 1 : 1 N : COOH ratios or above for **P1_SH_**,^18^ unstable particles were formed at a 1 : 0.5 N : COOH ratio for all the new peptides. Interestingly, **P2_SH_** showed the smallest range of formulation that yielded colloidally stable particles (Figure 3), suggesting again that this peptide may adopt a different conformation in solution. The deviation from the theoretical neutral point at N : COOH=1 : 1 towards B‐PEI‐rich mixtures had been previously described for other B‐PEI‐containing PIC nanoparticles,^29^ and it is likely a result of the incomplete protonation of all amines in B‐PEI due to Coulombic interactions between neighboring ammonium groups.^30^ Similarly, since peptides with higher multivalencies should have stronger affinities for B‐PEI, the exchange of the peptides with higher multivalency (i. e. **P3_SH_** and **P4_SH_**) between PIC nanoparticles and the solution should be slower than for the smaller peptides, thus trapping colloidally stable intermediates even at N : COOH ratios that should favor the formation of neutral PIC nanoparticles. Additionally, **P3_SH_** and **P4_SH_** showed no significant difference in the size or charge of any of the resulting PIC nanoparticles (Figure 3), suggesting that multivalency is the predominant factor that governs the self‐assembly of this collection of peptides. Regardless of the peptide used, no PIC nanoparticles could be detected at a 1 : 0.2 N : COOH ratio, probably as a result of the incomplete complexation of polyelectrolytes at this low concentration of peptide. The polydispersity indices (PDIs) of all PIC nanoparticles ranged between 0.01–0.06 with the exception of the formulations prepared at a 1 : 0.3 N : COOH ratio, which displayed significantly higher PDI values of up to 0.17 (Table S1). It was clear from dynamic light scattering (DLS) studies that formulations with lower peptide contents led to broader size distributions, although this phenomenon was less pronounced as the net charge of the peptide increased (Figure S6).

Size‐wise, **P2_SH_**–**P4_SH_** formulations showed a smaller variability in size than those reported for **P1_SH_** (Figure 3). Particles with larger hydrodynamic diameters (*D_H_*) were obtained for **P2_SH_** (*ca*. 300–400 nm) than for **P3_SH_** and **P4_SH_** (*ca*. 100–300 nm), regardless of the N : COOH ratio tested (Figure 3). Nanoparticles of smaller size for these two peptides could be the result of their higher multivalency (7 COOH groups for **P3_SH_** and **P4_SH_** *versus* 5 for **P2_SH_** and 3 for **P1_SH_**), which would result in more strongly and tightly bound polyelectrolyte networks within these nanoparticles. For all peptides, formulations prepared at a 1 : 0.3 N : COOH ratio gave the smallest PIC nanoparticles, with an average *D_H_* of *ca*. 110 nm and ζ‐potential of +19 mV – in agreement with the values previously found for **P1_SH_** at this N : COOH ratio.^18^

### Structural Characterization of PIC Particles

To further understand the impact that the multivalency and cross‐linking units of peptides **P1_SH_**–**P4_SH_** have on the structure of the PIC nanoparticles formed, these materials were characterized by Static Light Scattering (SLS). Multi‐angle SLS analysis of nanomaterials allows the calculation of their radius of gyration (*R_g_*) by deconvolution of the Zimm equation (*Eq*. 1);^31^,^32^ where *K* is a constant containing the optical parameters, *c* is the concentration of the sample, *R*_*θ*_ the Rayleigh ratio of the particles at a given scattering angle (*θ*), *q* is the scattering wave vector and *Mw_PIC_* is the average molecular weight of the nanoparticles. Thus, the ratio between gyration and hydrodynamic radii (i. e. *R_g_*/*R_H_*), calculated by SLS and DLS respectively, provides valuable information about the internal structure and shape of nanomaterials.^31^

(1) $$\frac{Kc}{R_{\theta }}=\frac{{Rg}^{2}}{3\;{Mw}_{PIC}}\;\times q^{2}+\frac{1}{{Mw}_{PIC}}$$

**Figure 4:**
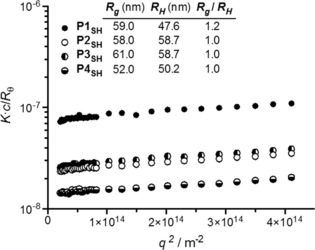
Partial Zimm plots obtained by SLS of filtered PIC particles prepared from peptides **P1_SH_**–**P4_SH_** at a 1 : 0.3 N : COOH ratio. The inset indicates the gyration (*R_g_*) and hydrodynamic (*R_H_*) radii simultaneously calculated by SLS and DLS, respectively.

PIC nanoparticles prepared from all four peptides at a 1 : 0.3 N : COOH ratio were selected for SLS analysis, being the most consistent formulation throughout the collection of peptides studied. The scattering intensity from a suspension of PIC nanoparticles was measured at different angles ranging from 20 to 100°, and their DLS profiles were simultaneously recorded. Unfortunately, non‐linear Zimm plots were obtained for all PIC particle samples with deviations at low angles, which made impossible the accurate measurement of *Mw_PIC_* and *R_g_* (Figure S7). Since larger particles have higher scattering contributions at low angles,^32^ we considered that this non‐linearity could be corrected by filtering the samples through membranes with a pore size of 0.45 μm, and thus remove any scatterers larger than the expected nanoparticles – which were all smaller than the filter's cut‐off size according to our previous DLS characterization (Figure 3). All filtered samples of PIC nanoparticles displayed linear Zimm plots that allowed the calculation of their *R_g_*, which was then compared with the *R_H_* obtained by DLS under the same conditions (Figure 4). The average *R_H_* measured from filtered samples was 57.5 nm, which is in agreement with the *D_H_* previously obtained from unfiltered nanoparticles (*ca*. 110 nm), suggesting that the filtration did not affect the main composition of PIC nanoparticles in the samples.

Nanoparticles prepared from peptides **P2_SH_**–**P4_SH_** consistently showed an *R_g_*/*R_H_* value of 1.0, whereas those containing the peptide **P1_SH_** presented a slight deviation towards *R_g_*/*R_H_* > 1, which is characteristic of anisotropic nanomaterials.^31^ This difference can be explained from the tendency of the latter nanoparticles to aggregate, as observed when their *D_H_* was monitored over time (Figure S8): Whereas PIC nanoparticles prepared from peptides **P2_SH_**–**P4_SH_** displayed the same size over time, the *D_H_* of the nanoparticles made from **P1_SH_** increased by 50% after 10 days, which was the time that passed between the preparation of these PIC nanoparticles and their SLS analysis. Nevertheless, the *R_g_*/*R_H_* values found for this collection of nanoparticles are in agreement with those reported in the literature for other PIC nanoparticles (1.0–1.6).^33^,^34^ These *R_g_*/*R_H_* ratios, which are higher than the value expected for a solid sphere (0.775),^31^ have been rationalized by the high polydispersity of PIC nanoparticles and their tendency to aggregate in some cases.^33^,^35^ Both of these factors act as a bias towards higher *R_g_*/*R_H_* values, making the elucidation of the internal structure of these nanomaterials extremely difficult.

In summary, the structural characterization of these nanoparticles by SLS and DLS indicates that all formulations have very similar *R_g_* and *R_H_* regardless of the peptide used, and therefore no distinct structural features should be expected in any of these complexes.

### Stability under Physiological Conditions of PIC Particles

**Figure 5:**
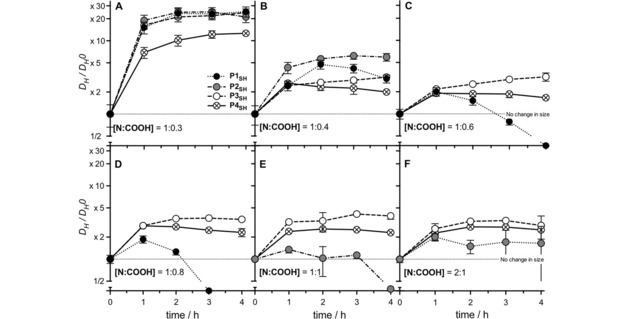
Relative change in size (*D_H_*/*D_H_0*) for PIC particles prepared from peptides **P1_SH_**–**P4_SH_** at representative N : COOH ratios under simulated physiological conditions (37 °C, 154 mM NaCl, pH 7.4) (**A** 1 : 0.3 [N : COOH] ratio, **B** 1 : 0.4 [N : COOH] ratio, **C** 1 : 0.6 [N : COOH] ratio, **D** 1 : 0.8 [N : COOH] ratio, **E** 1 : 1 [N : COOH] ratio and **F** 2 : 1 [N : COOH] ratio). Particle size (*D_H_*) was normalized to that of the PIC particles in the absence of NaCl (0 hours, No change in size). *n*=3, mean values±standard deviation. Results obtained directly after the assembly of the nanoparticles without prior filtration. Data for **P1_SH_** reproduced from I. Insua, E. Liamas, Z. Zhang, A. F. Peacock, A. M. Krachler, F. Fernandez‐Trillo, *Polym Chem* **2016**, *7*, 2684–2690 – Published by The Royal Society of Chemistry.

The electrostatic forces that keep PIC nanoparticles together can be shielded by small electrolytes, leading to swelling and ultimately breakdown of these nanoparticles.^36^ Hence, the integrity of PIC nanoparticles is often compromised by the concentrations of salts present in biological fluids. This lack of stability was the case for our previously reported nanoparticles, for which only one of the formulations was stable under simulated physiological conditions (Figure 5 and Figure S9, **P1_SH_**, 0.3 N : COOH ratio). The tolerance to physiological conditions of the new nanoparticles was evaluated by incubation in the presence of 154 mM NaCl at 37 °C, and the change of *D_H_* was monitored over four hours (Figure 5).

All PIC nanoparticles swelled when exposed to NaCl and this swelling was inversely proportional to the peptides’ multivalency and the degree of cross‐linking in the particle. As predicted, increasing the multivalency in the peptide resulted in tighter nanoparticles, that swelled less in the presence of NaCl (Figure 5). For all peptides, nanoparticles made at a 1 : 0.3 N : COOH ratio swelled the most, with particles prepared from **P2_SH_** and **P3_SH_** becoming over 20 times bigger after 4 hours of incubation (Figure 5A), a similar increase in size to that reported for **P1_SH_**. Interestingly, those prepared from **P4_SH_**, which carries an extra cysteine and should give higher cross‐linking densities at the same N : COOH ratio, were the least affected by this saline medium, regardless of the formulation employed (Figure 5, ⊗).

Overall, all the particles prepared with the new peptides **P2_SH_**–**P4_SH_** showed better physiological stabilities than those previously reported for trivalent **P1_SH_** (Figure 5, •),^18^ reinforcing the correlation between peptide multivalency and PIC nanoparticle stability (Figure 5 and Figure S9). Particles prepared from the least multivalent peptide **P2_SH_** showed more polydisperse aggregates after 2 and 3 hours and, for those prepared at a 1 : 1 N : COOH ratio, no particles could be detected after 4 hours of incubation under these conditions (Figure 5E, •). These results highlight the critical effect that multivalency and cross‐linking degree have on the salt tolerance of PIC particles, as evidenced by their physiological stability: **P1_SH_**<**P2_SH_**<**P3_SH_**<**P4_SH_**.

### Stability Upon Freeze‐drying

Finally, we evaluated if the increase in stability observed under physiological conditions for the nanoparticles formed with the new peptides **P2_SH_**–**P4_SH_** could be correlated to an increased stability upon storage. Nanoparticles prepared at a 1 : 0.3 N : COOH ratio were selected for this evaluation, being a consistent formulation across all peptides evaluated. Also, nanoparticles prepared at this N : COOH ratio have the least amount of peptide and while stable, swelled more upon incubation in physiological conditions. This swelling may indicate that the cohesive forces in the core of these nanoparticles are weak enough to accommodate the presence of competing counterions, or a higher tendency to aggregate, making it an ideal formulation to test stability. To this end, freshly prepared nanoparticles at this N : COOH ratio were allowed to stand in a dark, cool and dry place, and their size monitored over time (Figure S8). As described before, no change in size was observed for any the nanoparticles prepared with the new peptides **P2_SH_**–**P4_SH_**, even after 10 days of storage. This was not the case for the nanoparticles prepared with **P1_SH_** which quickly increased in size.

**Figure 6:**
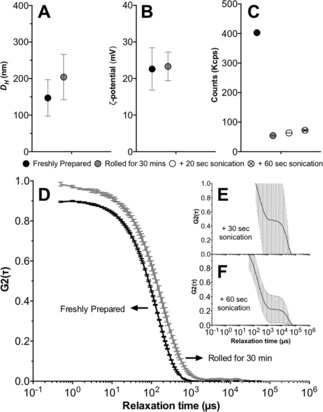
Effect of freeze‐drying and resuspension of the size (A), ζ‐potential (B) and number of counts (C) of nanoparticles prepared with **P3_SH_** at a 0.3 N : COOH ratio. Autocorrelation function curves for freshly prepared and nanoparticles following resuspension on a roller for 30 mins (D) and additional sonication for 30 sec (E) or 60 secs (F).

Nanoparticles prepared from **P3_SH_** at a 0.3 N : COOH ratio, which showed excellent stability in solution, were then selected as a representative example to evaluate the potential of these formulations to be stored as a powder. Therefore, freshly prepared nanoparticles from **P3_SH_** at a 0.3 N : COOH ratio were freeze‐dried overnight to yield a white powder. Approximately 1.32 mg of powder were recovered for all 3 samples prepared (standard deviation 0.042), in close agreement with the 1.47 mg expected taking into account the amount of peptide and B‐PEI used, and how much solid content was in the buffer used to prepare the nanoparticle suspension. This powder was then reconstituted in deionized water to give the original volume (1 mL). Samples were then gently resuspended on a roller for 30 mins and characterized via DLS and ζ‐potential. A small increase in size was observed following resuspension (Figure 6A, •) in agreement with the small shift in the autocorrelation curve (Figure 6D), while no changes in the ζ‐potential of the particles were observed following resuspension on the rollers (Figure 6B, •). A bigger effect was observed on the number of counts, which decreased from 402.8±2.3 to 54.2±1.2 (Figure 6C, •) suggesting that less particles were available in suspension following reconstitution.

These samples were then sonicated for 30 or 60 seconds to try to increase the number of nanoparticles in suspension. While a small increase in the number of counts was observed (Figure 6C, ○ and ⊗ for 30 and 60 secs respectively), sonication had a detrimental effect on the nanoparticles and very noisy autocorrelation function curves were obtained (Figure 6E and F), which prevented accurate characterization of their size and ζ‐potential (Figure 6A and B).

## Conclusions

Three new enzyme‐responsive anionic peptides (**P2_SH_**–**P4_SH_**) have been synthesized and their use in the preparation of PIC nanoparticles containing the antimicrobial polymer B‐PEI reported. These peptides were designed to incorporate the LasB‐degradable sequence −GLA−, and increasing amounts of glutamic acids for a stronger interaction with B‐PEI (**P2_SH_** and **P3_SH_**), and an additional cysteine (**P4_SH_**) to generate PIC nanoparticles with higher cross‐linking density. The enzymatic degradation of these peptides was assessed against bacterial (LasB) and human (HLE) elastases and, while all peptides were degraded by the bacterial elastase, no direct correlation with the multivalency of the peptides could be identified. All new peptides formed PIC nanoparticles when incubated with B‐PEI, and a broad range of formulations could be accessed by varying the ratio of polyelectrolytes (i. e. N : COOH ratio). Our results show that these new peptides allow the preparation of negatively charged nanoparticles, not previously accessible using the less multivalent peptide **P1_SH_**, previously reported by our group.^18^ More importantly, the stability of the new nanoparticles under simulated physiological conditions increased with increasing multivalency and cross‐linking degree of the peptides (i. e. **P2_SH_**<**P3_SH_**<**P4_SH_**) and displayed higher saline stability than those prepared using **P1_SH_**. The structural analysis of these peptides and their resulting PIC nanoparticles by CD and SLS, respectively, suggests that there are no differences in conformation or architecture across the whole collection of materials tested that could explain these differences in susceptibility against LasB and stability. Finally, the stability of representative formulations upon storage was evaluated. Our results demonstrate the importance of carefully optimizing peptide sequence and multivalency in the design of peptide‐based PIC particles and we believe these new peptides and formulations will underpin the future development of “smart” delivery systems for antimicrobials. Our efforts to identify LasB‐responsive nanoparticles with optimized release and antimicrobial activity, as well as the application of these peptides for the preparation of other delivery systems, will be reported in due course.

## **Experimental Section**

### Materials

Enzymes (*Pseudomonas aeruginosa* Elastase (LasB): EC 3.4.24.26 and Human Leucocyte Elastase (HLE): EC 3.4.21.37) were purchased from Merck Millipore. Branched poly(ethylene imine) 25 kDa average molecular weight (B‐PEI) and 4‐(2‐hydroxyethyl)piperazine‐1‐ethanesulfonic acid (HEPES) were bought from Sigma‐Aldrich®. Fluorescamine and dimethylsulphoxide (DMSO) were purchased from Acros Organics^TM^. Ethylenediaminetetraacetic acid (EDTA) was purchased from Alfa Aesar®. Nylon 0.45 μm syringe filters were purchased from Camlab.

### Instrumentation

Dynamic Light Scattering (DLS) and ζ‐potential measurements were carried out with a Zetasizer Nano ZSP (Malvern Instruments Ltd.) stabilized at 37 °C. DLS was read at 173° (backscattering) for 60 seconds in triplicate and ζ‐potentials were averaged from 30 measurements at 140 V. DLS correlograms were processed with Malvern's *General Purpose* non‐negative least squares algorithm, and their *D_H_* values correspond to the mean size and standard deviation of the only population found in their size‐intensity plots. A FLUOstar Omega (BMGLabtech Gmbh) microplate reader was used to incubate and measure fluorescamine reactions. Static Light Scattering (SLS) and simultaneous DLS data was collected using an CGS‐3 Compact Goniometer System (ALV Gmbh) stabilized at 20 °C and operating at a wavelength of 633 nm against a toluene standard.

### Preparation of PIC Nanoparticles

For nanoparticles prepared at a 1 : 0.3 N : COOH ratio (defined as the ratio between amines in B‐PEI and carboxylic acids in the peptides), stock solutions of B‐PEI (2.5 mM in amines) and peptide (**P1_SH_**–**P4_SH_**) (0.75 mM in carboxylate groups) in 5 mM HEPES buffer at pH 7.4 were prepared. Then, both solutions were filtered and mixed in equal volumes drop‐wise under stirring. The reaction mixture was stirred at room temperature for 24 hours open to air to allow thiol oxidation. PIC nanoparticles prepared at different N : COOH ratios were obtained by changing the concentration of peptide stock solution and mixing with the 2.5 mM B‐PEI stock following the same protocol (Table S1). After 24 hours, samples were analyzed directly by DLS and ζ‐potential without prior filtration.

### Enzymatic Degradation of Peptides – Fluorescamine Assay

Stock solutions of peptide (1 mM) or succinyl casein (0.5 mg/mL) were prepared in 25 mM Na_2_B_4_O_7_ buffer at pH 8.0 with 10 mM CaCl_2_ and 10% v/v DMSO. 125 μL of these substrate solutions were added to a 96‐well black‐walled microplate and mixed with 125 μL of the same buffer without DMSO, containing 15 μg of enzyme (LasB or HLE). Solutions of enzymes and substrates alone were prepared as controls. Every sample was prepared in triplicate. The microplate was incubated at 37 °C for 4 hours under orbital shaking. After 4 hours, 50 μL of 0.1 M EDTA in water at pH 8.0 were added to each well to quench all enzymatic activity. Then, each sample was mixed in a 1 : 1 volume ratio with a 1 mM solution of fluorescamine^19^ in methanol. The microplate was incubated at 37 °C under orbital shaking for 30 minutes. After this time, fluorescence was measured exciting at 355 nm and reading the emission at 460±10 nm.

### Stability of PIC Nanoparticles in Simulated Physiological Conditions

182 μL of a 1 M solution of NaCl in water was added to a sample of PIC nanoparticles (1 mL), prepared as described above. This mixture was then incubated at 37 °C to obtain physiological osmotic pressure and temperature. Every hour, the sample was analyzed by DLS as described above.

### Freeze‐drying and Resuspension of PIC Nanoparticles

A sample (1 mL) of freshly made PIC nanoparticles from **P3_SH_** and B‐PEI at a 1 : 0.3 N : COOH ratio was frozen inside a tared 2 mL‐scintillation vial, to be then left overnight under vacuum at −80 °C. A white solid was thus obtained, which was weighed by difference inside the tared vial. Then, 1 mL of deionized water was added to the vial and the mixture was gently and simultaneously rocked and rolled for 30 min. After this time, the sample was characterized by DLS and ζ‐potential as indicated above. Replicates of this freeze‐dried and reconstituted sample were further processed by immersion in an ultrasonic bath at room temperature for 30 and 60 sec, and characterized likewise. For the measurement of light scattering intensity by DLS (i. e. counts, Figure 6C), the attenuator value of the instrument was kept constant across all measurements.

## Author Contributions

All authors contributed to the experimental set‐up and discussed the results. II and MP synthesized and characterized the peptides. II and FFT designed the peptides, and the nanoparticle preparation and characterization. II, FFT and AMK designed the enzymatic assays. LDB, RK, APB and RKOR designed and performed structural characterization. II carried out all other experiments. FFT and AMK secured funding. II and FFT analyzed the data and wrote the paper, with all other authors contributing to the final version of the manuscript.

## Conflict of interest

The authors declare no conflict of interest.

## Supporting information

As a service to our authors and readers, this journal provides supporting information supplied by the authors. Such materials are peer reviewed and may be re‐organized for online delivery, but are not copy‐edited or typeset. Technical support issues arising from supporting information (other than missing files) should be addressed to the authors.

Supplementary

## References

[1] A. D. Kulkarni , Y. H. Vanjari , K. H. Sancheti , H. M. Patel , V. S. Belgamwar , S. J. Surana , C. V. Pardeshi , Artif. Cells, Nanomed., Biotechnol. 2016, 44, 1615–1625. 26757773 10.3109/21691401.2015.1129624

[2] D. V. Pergushov , A. H. E. Müller , F. H. Schacher , Chem. Soc. Rev. 2012, 41, 6888–6901. 22814675 10.1039/c2cs35135h

[3] I. Insua , A. Wilkinson , F. Fernández-Trillo , Eur. Polym. J. 2016, 81, 198–215. 27524831 10.1016/j.eurpolymj.2016.06.003 PMC4973809

[4] S. C. How , Y. F. Chen , P. L. Hsieh , S. S. S. Wang , J.-S. Jan , Colloids Surf. B 2017, 153, 244–252. 10.1016/j.colsurfb.2017.02.032 28267669

[5] L. Zhang , J. Wang , C. Ni , Y. Zhang , G. Shi , Mater. Sci. Eng. C 2016, 58, 724–729. 10.1016/j.msec.2015.09.044 26478364

[6] L. Yang , S. Gao , S. Asghar , G. Liu , J. Song , X. Wang , Q. Ping , C. Zhang , Y. Xiao , Int. J. Biol. Macromol. 2015, 72, 1391–1401. 25450553 10.1016/j.ijbiomac.2014.10.039

[7] Y.-H. Hsieh , Y.-T. Hsiao , J.-S. Jan , Soft Matter 2014, 10, 9568–9576. 25357089 10.1039/c4sm02033b

[8] I. Insua , S. Majok , A. F. A. Peacock , A. M. Krachler , F. Fernández-Trillo , Eur. Polym. J. 2017, 87, 478–486. 28280277 10.1016/j.eurpolymj.2016.08.023 PMC5327956

[9] K. A. Les , A. H. A. Mohamed-Ahmed , S. Balan , J.-W. Choi , D. Martin , V. Yardley , K. Powell , A. Godwin , S. J. Brocchini , S. Brocchini , Polym. Chem. 2014, 5, 1037–1048.

[10] K. L. Niece , A. D. Vaughan , D. I. Devore , J. Biomed. Mater. Res. A 2013, 101, 2548–2558. 23364909 10.1002/jbm.a.34555

[11] I. Insua , L. Zizmare , A. F. A. Peacock , A. M. Krachler , F. Fernández-Trillo , Sci. Rep. 2017, 7, 9396–10. 28839223 10.1038/s41598-017-09667-3 PMC5570901

[12] A. Carmona-Ribeiro , J. Sampaio , H. Santos , L. Carrasco , Drug Delivery Letters 2017, 7, 39–47.

[13] E. F. Craparo , B. Porsio , D. Schillaci , M. G. Cusimano , D. Spigolon , G. Giammona , G. Cavallaro , Nanomedicine 2017, 12, 25–42. 27879162 10.2217/nnm-2016-0262

[14] D. Vehlow , R. Schmidt , A. Gebert , M. Siebert , K. S. Lips , M. Müller , Nanomaterials 2016, 6, 53. 10.3390/nano6030053 PMC5302517 28344311

[15] U. Lächelt , E. Wagner , Chem. Rev. 2015, 115, 11043–11078. 25872804 10.1021/cr5006793

[16] R. V. Ulijn , J. Mater. Chem. 2006, 16, 2217–2225.

[17] W. E. Kaman , J. P. Hays , H. P. Endtz , F. J. Bikker , Eur. J. Clin. Microbiol. Infect. Dis. 2014, 33, 1081–1087. 24535571 10.1007/s10096-014-2075-1

[18] I. Insua , E. Liamas , Z. Zhang , A. F. A. Peacock , A. M. Krachler , F. Fernández-Trillo , Polym. Chem. 2016, 7, 2684–2690. 27148427 10.1039/c6py00146g PMC4841106

[19] S. Udenfriend , S. Stein , P. Böhlen , W. Dairman , W. Leimgruber , M. Weigele , Science 1972, 178, 871–872. 5085985 10.1126/science.178.4063.871

[20] K. Morihara , H. Tsuzuki , Arch. Biochem. Biophys. 1971, 146, 291–296. 5004124 10.1016/s0003-9861(71)80066-8

[21] S. Suter , U. B. Schaad , L. Roux , U. E. Nydegger , F. A. Waldvogel , J. Infect. Dis. 1984, 149, 523–531. 6427360 10.1093/infdis/149.4.523

[22] N. J. Greenfield , Nat. Protoc. 2006, 1, 2876–2890. 17406547 10.1038/nprot.2006.202 PMC2728378

[23] B. Korkmaz , S. Attucci , T. Moreau , E. Godat , L. Juliano , F. Gauthier , Am. J. Respir. Cell Mol. Biol. 2004, 30, 801–807. 14693667 10.1165/rcmb.2003-0139OC

[24] B. Korkmaz , S. Attucci , E. Hazouard , M. Ferrandière , M. L. Jourdan , M. Brillard-Bourdet , L. Juliano , F. Gauthier , J. Biol. Chem. 2002, 277, 39074–39081. 12114510 10.1074/jbc.M202918200

[25] K. Morihara , H. Tsuzuki , T. Oka , H. Inoue , M. Ebata , J. Biol. Chem. 1965, 240, 3295–3304. 14321366

[26] B. Wretlind , T. Wadström , J. Gen. Microbiol. 1977, 103, 319–327. 413876 10.1099/00221287-103-2-319

[27] E. Kessler , D. E. Ohman , in Handbook of Proteolytic Enzymes, Elsevier, 2013, pp. 582–592.

[28] M. Müller , Polyelectrolyte Complexes in the Dispersed and Solid State II, Springer, Berlin, 2013.

[29] H. Sato , A. Nakajima , Polym. J. 1975, 7, 241–247.

[30] M. Borkovec , G. Koper , Macromolecules 1997, 30, 2151–2158.

[31] J. P. Patterson , M. P. Robin , C. Chassenieux , O. Colombani , R. K. O′Reilly , Chem. Soc. Rev. 2014, 43, 2412–2425. 24519401 10.1039/c3cs60454c

[32] B. H. Zimm , J. Chem. Phys. 1948, 16, 1099–1116.

[33] K. Ueno , H. Ueno , T. Sato , Polym. J. 2012, 44, 59–64.

[34] D. Fischer , H. Dautzenberg , K. Kunath , T. Kissel , Int. J. Pharm. 2004, 280, 253–269. 15265564 10.1016/j.ijpharm.2004.05.018

[35] H. Dautzenberg , J. Kriz , Langmuir 2003, 19, 5204–5211.

[36] H. Dautzenberg , G. Rother , Macromol. Chem. Phys. 2004, 205, 114–121.

