# Supplementary material for: Structural Determinants of the Stability of Enzyme‐Responsive Polyion Complex Nanoparticles Targeting Pseudomonas aeruginosa’s Elastase
Source: ChemNanoMat. 2018 Apr 23;4(8):807–14. doi: 10.1002/cnma.201800054 (PMC6146907; doi:10.1002/cnma.201800054)
Supplement: Supplementary file 1 — Supplementary [file CNMA-4-807-s001.pdf]

Supporting Information

© Copyright Wiley-VCH Verlag GmbH & Co. KGaA, 69451 Weinheim, 2018

Structural Determinants of the Stability of Enzyme-Responsive Polyion Complex Nanoparticles Targeting *Pseudomonas aeruginosa*'s Elastase

Ignacio Insua, Marion Petit, Lewis D. Blackman, Robert Keogh, Anaïs Pitto-Barry, Rachel K. O'Reilly, Anna F. A. Peacock, Anne Marie Krachler, and Francisco Fernandez-Trillo*© 2018 The Authors. Published by Wiley-VCH Verlag GmbH & Co. KGaA.
This is an open access article under the terms of the Creative Commons Attribution License, which permits use, distribution and reproduction in any medium, provided the original work is properly cited.This manuscript is part of a Special Issue on Supramolecular Nanostructures.
.

Structural determinants of the stability of enzyme-responsive polyion complex (PIC) nanoparticles targeting *Pseudomonas aeruginosa*'s elastase

Ignacio Insua,[a],[b] Marion Petit,[a] Lewis D. Blackman,[c] Robert Keogh,[c] Anaïs Pitto-Barry,[c] Rachel K. O'Reilly,[c] Anna F. A. Peacock,[a] Anne Marie Krachler,[b],[d] and Francisco Fernandez-Trillo*[a],[b]

[a] School of Chemistry, University of Birmingham, Edgbaston, B15 2TT, UK. [b] Institute of Microbiology and Infection – School of Biosciences, University of Birmingham, Edgbaston, B15 2TT, UK. [c] School of Chemistry, University of Warwick, Coventry, CV4 7AL, UK. [d] Current address: Department of Microbiology and Molecular Genetics, University of Texas Health Science Center, 6431 Fannin. Houston (TX), USA

**Email: f.fernandez-trillo@bham.ac.uk*

1. Materials 2
2. Instrumentation 2
3. Peptide synthesis 2
4. Peptide characterisation 2
5. Enzymatic degradation of peptides 6
6. Circular dichroism characterisation of peptides 7
7. PIC particle characterisation 8
8. SLS characterisation of unfiltered PIC particles 9
9. Shelf stability of PIC particles 10
10. Stability of the particles under physiological conditions 10
11. Additional references 11

1. Materials

N-Fmoc-protected L-amino acids were purchased from Merck Millipore. *N*,*N*-dimethylformamide (DMF), piperidine 20% v/v in DMF and acetic anhydride were purchased from Sigma-Aldrich®. H-L-Cys(Trt)-2-chlorotrityl resin (0.49 mmol/g) was bought from AGTC Bio Products Ltd. *N,N*-diisopropylethylamine (DIPEA), triisopropylsilane (TIPS), 1,2-ethanedithiol (EDT) and trifluoroacetic acid (TFA) were bought from Alfa Aesar®. *N*,*N*,*N*',*N*'-tetramethyl-*O*-(1*H*-benzotriazol-1-yl)uronium hexafluorophosphate (HBTU) was purchased from Carbosynth Ltd. All other chemicals were purchased from Fisher Scientific UK Ltd. and were used without further purification.

2. Instrumentation

NMR data was acquired on a Bruker Avance III operating at 400 MHz and fitted with a 5 mm DUL probe ($^{1}H/^{13}C$). MS spectra were obtained on a Xevo® G2-XS ToF (Waters) from electrospray ionisation (ESI) and time-of-flight (TOF) measurement in positive ion mode. High-resolution MS data was calculated by comparison with leucine-enkephalin as internal standard. Reverse phase (RP) HPLC analysis was run through a Kinetex® C18-EVO column (Phenomenex®): 5 µm, 100 Å, 250x4.60 mm. A gradient from 3 to 20% of (CH_3CN + 0.05% TFA) in (H_2O + 0.05% TFA) was used at 1 mL/min. The column was maintained at 35°C and UV-VIS detection was set at 210 nm. Circular dichroism spectra were recorded in 1 mm path length quartz cuvettes on a Jasco J-715 spectropolarimeter. The observed ellipticity in millidegrees was converted into mean residue ellipticity and it is reported in units of degree $\cdot$ $dmol^{-1} \cdot cm^{2} \cdot residue^{-1}$.[1]

3. Peptide synthesis

500 mg of H-L-Cys(Trt)-2-chlorotrityl resin (0.245 mmol) were swollen in 5 mL of DMF for 30 minutes. Then, solutions of Fmoc-L-amino acid (3 eq), HBTU (2.8 eq) and DIPEA (2.8 eq) in DMF were added to a final volume of 5 mL. The reaction mixture was rolled for 1 h at room temperature, after which a negative chloranil test[2] indicated the reaction had gone to completion. The *N*-terminal Fmoc protecting group was removed by washing the resin with 5 mL of piperidine 20% v/v in DMF during 10 minutes. A positive chloranil test confirmed the removal of the Fmoc group, and the previous steps were repeated to couple all amino acids in the sequence. Following the coupling of the last amino acid, the Fmoc group was removed and the terminal amine of the peptide was capped by reacting with 5 mL of acetic anhydride:DIPEA:DMF (1:1:3) for 1 h at room temperature. Then, the resin was thoroughly washed with DMF and diethyl ether, and the peptide was cleaved from the resin with 5 mL of a mixture containing TFA (80%), TIPS (8%) and EDT (12%) for 2 h. After this time, the solution was concentrated under argon and precipitated in chilled diethyl ether:hexane (1:4). Finally, the suspension was centrifuged and the pellets were washed twice with diethyl ether:hexane (1:4), dissolved in water and freeze-dried. Peptides that showed impurities by ^{1}H-NMR (*e.g.* trityl protecting groups) were further purified by dissolving the material in 2 mL of a 10% TIPS solution in TFA and precipitating in chilled diethyl ether:hexane (1:4), to be then centrifuged and freeze-dried as explained above. Peptide purity was determined by HPLC.

4. Peptide characterisation

The synthesis and characterisation of peptides $\mathbf{P1_{SH}}$ (Ac-CEGLAEC-OH) and **P5** (H_2N-LAE-OH) has been previously reported.[3]

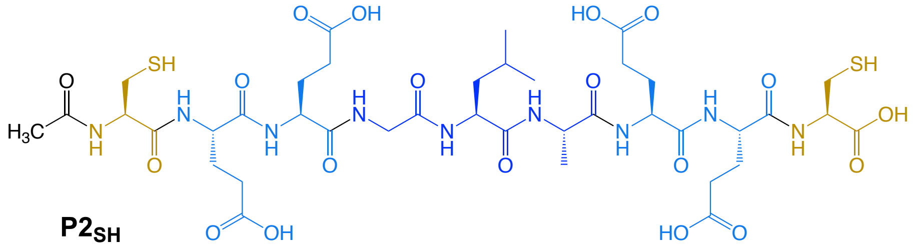

P2$_{SH}$

P2$_{SH}$, Ac-CEEGLAEEC-OH (154.8 mg, 62% yield) **^{1}H-NMR** (400 MHz, DMSO-*d6*): δ 0.84(dd, *J*=15.0, 6.5 Hz, 6H, H^{δ}-Leu); 1.20(d, *J*=7.1 Hz, 3H, H^{β}-Ala 1.35-1.47(m, 2H, H^{β}-Leu); 1.50-1.63(m, 1H, H^{γ}-Leu); 1.66-1.80(m, 4H, H^{β}-Glu); 1.84-1.96(m, 4H, H^{β}-Glu); 1.87(s, 3H, Ac); 2.16-2.31(m, 8H, H^{γ}-Glu); 2.36(t, *J*=8.5 Hz, 1H, SH); 2.45(t, *J*=8.6 Hz, 1H, SH); 2.62-2.89(m, 4H, Hβ-Cys); 3.70(ddd, J=56.8, 16.7, 5.7 Hz, 2H, Hα-Gly); 4.20-4.41(m, 8H, Hα); 7.89(d, J=8.1 Hz, 1H, NHCO); 7.92(d, J=8.5 Hz, 1H, NHCO); 7.95(d, J=7.7 Hz, 1H, NHCO); 7.98(d, J=7.7 Hz, 1H, NHCO); 8.11-8.22(m, 5H, NHCO); 12.20(br.s, 4H, COOH) ppm. 13C-NMR (400 MHz, DMSO-d6): δ 17.4(Cβ-Ala); 21.4(Hδ-Leu); 22.3(Ac); 22.9(Hδ-Leu); 23.9(Hγ-Leu); 25.2(Cβ-Cys); 25.9(Cβ-Cys); 26.7(Cβ-Cys); 27.2(Cβ-Glu); 29.7(Cγ-Glu); 29.8(Cγ-Glu); 29.8(Cγ-Glu); 29.9(Cγ-Glu); 40.7(Cβ-Leu); 41.6(Cα-Gly); 48.0(C^{α}); 50.6(C^{α}); 51.4(C^{α}); 51.5(C^{α}); 51.8(C^{α}); 52.0(C^{α}); 54.2(C^{α}); 55.0(C^{α}); 168.5(NHCO); 169.8(Ac); 170.2(NHCO); 170.9(NHCO); 171.0(NHCO); 171.1(NHCO); 171.3(NHCO); 171.4(NHCO); 171.9(NHCO); 172.2(NHCO); 174.0(COOH) ppm. **MS** (ESI-TOF, +eV): m/z 1024.4 $[M+H]^{+}$; 531.7 $[M+Ca]^{2+}$. **HR-MS** (ESI-TOF, +eV): m/z 1024.3603 (calculated for $[M+H]^{+}$); 1024.3605 (found). Purity by **HPLC** = 92% (Rt = 24.2 min).

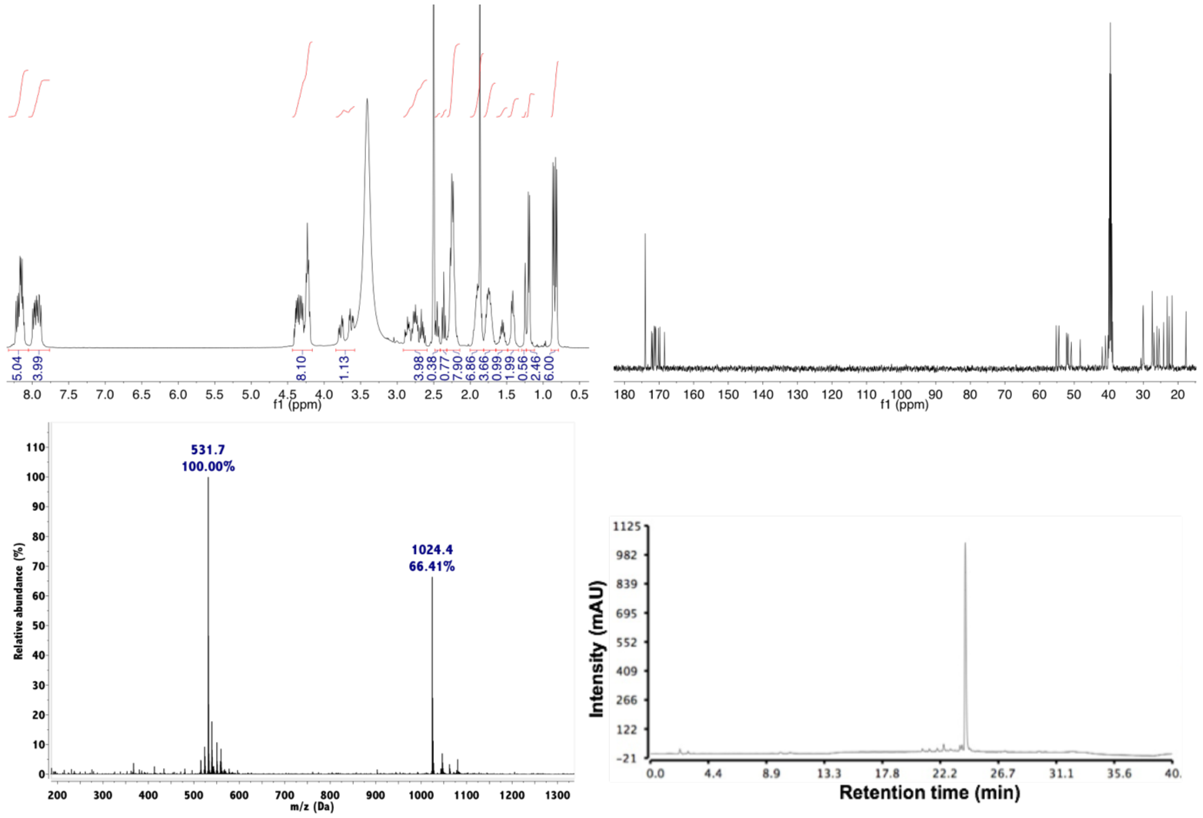

Figure S1 Characterisation of peptide **P2$_{SH}$** (Ac-C-EE-GLA-EE-C-OH). Top: ^{1}H (left) and ^{13}C (right) NMR spectra (400 MHz, DMSO-*d$_6$*). Bottom: Mass spectrum (left) and RP-HPLC chromatogram (right).

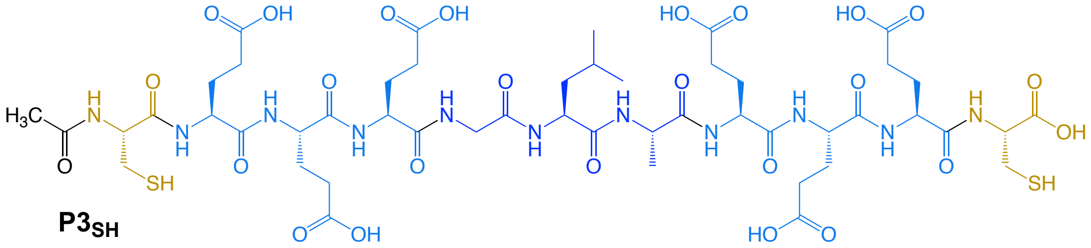

P3$_{SH}$

P3$_{SH}$, Ac-CEEEGLAEEEC-OH (152.5 mg, 72% yield) ^{1}**H-NMR** (400 MHz, DMSO-*d6*): δ 0.84(dd, *J*=14.8, 6.4 Hz, 6H, H^{δ}-Leu); 1.19(d, *J*=7.0 Hz, 3H, H^{β}-Ala); 1.36-1.47(m, 2H, H^{β}-Leu); 1.50-1.63(m, 1H, H^{γ}-Leu); 1.67-1.81(m, 6H, H^{β}-Glu); 1.84-1.95(m, 6H, H^{β}-Glu); 1.87(s, 3H, Ac); 2.17-2.29(m, 12H, H^{γ}-Glu); 2.34(t, *J*=8.5 Hz, 1H, SH); 2.44(t, *J*=8.6 Hz, 1H, SH); 2.62-2.89(m, 4H, H^{β}-Cys); 3.70(ddd, *J*=75.2, 16.7, 5.7 Hz, 2H, H^{α}-Gly); 4.19-4.42(m, 10H, H^{α}); 7.89-7.94(m, 4H, NHCO); 8.04(d, *J*=7.4 Hz, 1H, NHCO); 8.08(d, *J*=7.6 Hz, 1H, NHCO); 8.11-8.20(m, 5H, NHCO); 12.17(br.s, 5H, COOH) ppm. ^{13}**C-NMR** (400 MHz, DMSO-*d6*): δ 17.4(C^{β}-Ala); 21.4(H^{δ}-Leu); 22.3(Ac); 22.8(H^{δ}-Leu); 23.9(H^{γ}-Leu); 25.2(C^{β}-Cys); 25.9(C^{β}-Cys); 26.7(C^{β}-Cys); 27.0(C^{β}-Glu); 27.1(C^{β}-Glu); 27.2(C^{β}-Glu); 29.9 (C^{γ}-Glu); 40.8(C^{β}-Leu); 41.6(C^{α}-Gly); 48.0(C^{α}); 50.6(C^{α}); 51.5(C^{α}); 51.6(C^{α}); 51.7(C^{α}); 51.8(C^{α}); 51.9(C^{α}); 54.2(C^{α}); 54.9(C^{α}); 168.5(NHCO); 169.7(Ac); 170.1(NHCO); 171.0(NHCO); 171.0(NHCO); 171.2(NHCO); 171.4(NHCO); 171.4(NHCO); 171.9(NHCO); 172.3(NHCO); 174.0(COOH) ppm. **MS** (ESI-TOF, +eV): m/z 1282.4 $[M+H]^{+}$; 668.7 $[M+H+Na+CH_3OH]^{2+}$; 660.7 $[M+Ca]^{2+}$; 641.7 $[M+2H]^{2+}$. **HR-MS** (ESI-TOF, +eV): m/z 1282.4455 (calculated for $[M+H]^{+}$); 1282.4452 (found). Purity by **HPLC** = 90% (Rt = 23.8 min).

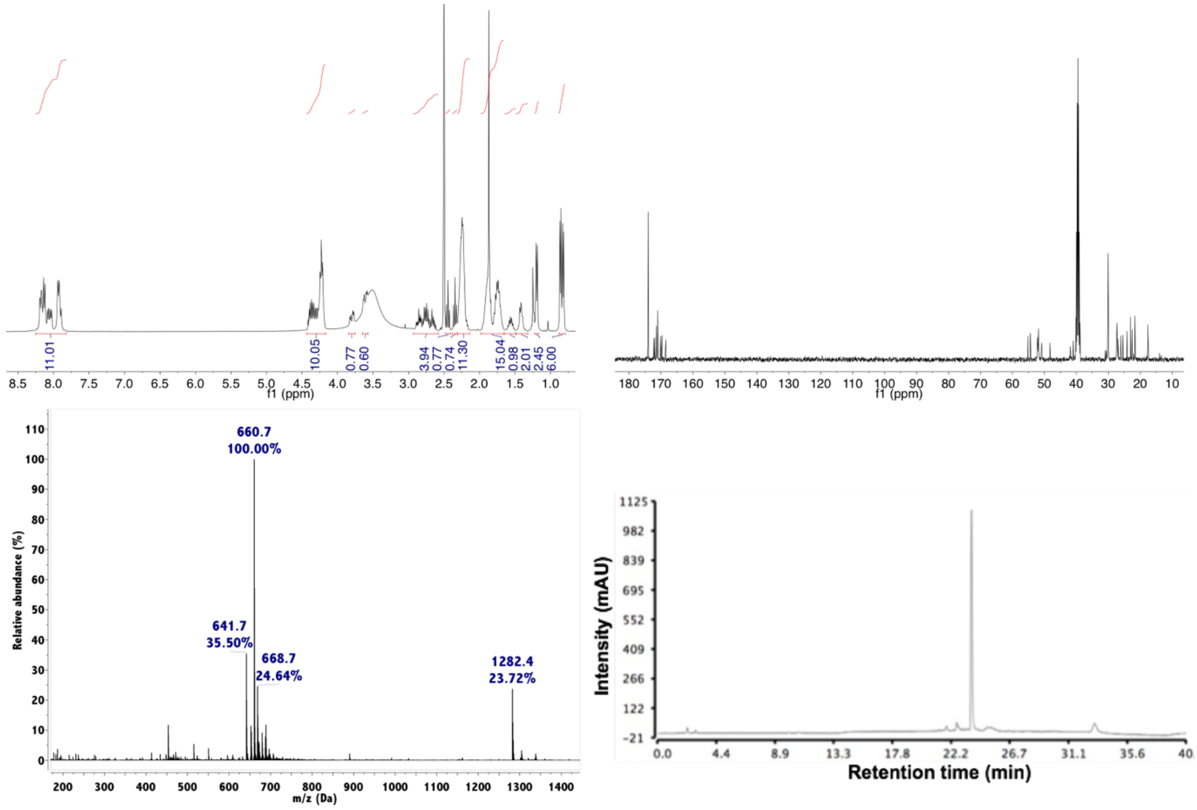

Figure S2 Characterisation of peptide **P3$_{SH}$** (Ac-C-EEE-GLA-EEE-C-OH). Top: ^{1}H (left) and ^{13}C (right) NMR spectra (400 MHz, DMSO-*d$_6$*). Bottom: Mass spectrum (left) and RP-HPLC chromatogram (right).

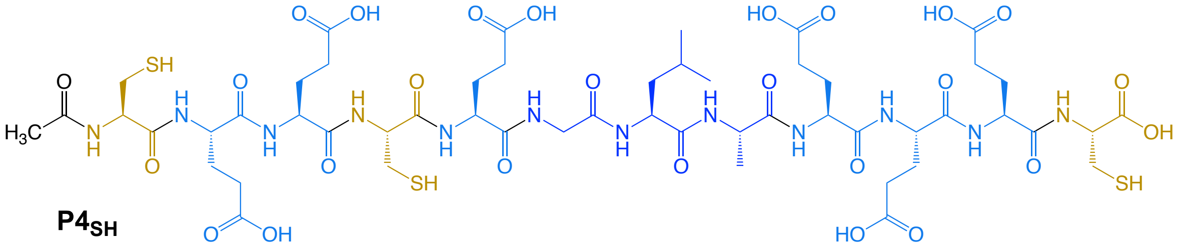

P4$_{SH}$

P4$_{SH}$, Ac-CEECEGLAEEEC-OH (208.0 mg, 98% yield) **^{1}H-NMR** (400 MHz, DMSO-*d6*): δ 0.84(dd, *J*=14.5, 6.6 Hz, 6H, H$^{\delta}$-Leu); 1.19(d, *J*=7.0 Hz, 3H, H$^{\beta}$-Ala); 1.36-1.46(m, 2H, H$^{\beta}$-Leu); 1.51-1.61(m, 1H, H$^{\gamma}$-Leu); 1.67-1.81(m, 6H, H$^{\beta}$-Glu); 1.83-1.95(m, 6H, H$^{\beta}$-Glu); 1.87(s, 3H, Ac); 2.17-2.31(m, 12H, H$^{\gamma}$-Glu); 2.36(dt, *J*=8.5, 2.8 Hz, 2H, SH); 2.44(t, *J*=8.5 Hz, 1H, SH); 2.62-2.89(m, 6H, H$^{\beta}$-Cys); 3.70(ddd, *J*=54.5, 16.7, 5.8 Hz, 2H, H$^{\alpha}$-Gly); 4.19-4.42(m, 11H, H$^{\alpha}$); 7.90-8.20(m, 12H, NHCO); 12.16(br.s, 5H, COOH) ppm. **^{13}C-NMR** (400 MHz, DMSO-*d6*): δ 17.4(C$^{\beta}$-Ala); 21.4(H$^{\delta}$-Leu); 22.3(Ac); 22.9(H$^{\delta}$-Leu); 23.9(H$^{\gamma}$-Leu); 25.2(C$^{\beta}$-Cys); 25.9(C$^{\beta}$-Cys); 26.8(C$^{\beta}$-Cys); 27.0(C$^{\beta}$-Glu); 27.1(C$^{\beta}$-Glu); 27.1(C$^{\beta}$-Glu); 27.2(C$^{\beta}$-Glu); 40.8(C$^{\beta}$-Leu); 41.6(C$^{\alpha}$-Gly); 48.0(C$^{\alpha}$); 50.5(C$^{\alpha}$); 51.5(C$^{\alpha}$); 51.6(C$^{\alpha}$); 51.7(C$^{\alpha}$); 51.9(C$^{\alpha}$); 52.1(C$^{\alpha}$); 54.2(C$^{\alpha}$); 54.8(C$^{\alpha}$); 54.9(C$^{\alpha}$); 168.4(NHCO); 169.7(Ac); 170.1(NHCO); 171.0(NHCO); 171.0(NHCO); 171.1(NHCO); 171.1(NHCO); 171.2(NHCO); 171.3(NHCO); 171.4(NHCO); 171.9(NHCO); 172.2(NHCO); 174.0(COOH) ppm. **MS** (ESI-TOF, +eV): m/z 1407.4 $[M+Na]^{+}$; 1385.5 $[M+H]^{+}$; 712.2 $[M+Ca]^{2+}$; 704.2 $[M+H+Na]^{2+}$; 693.2 $[M+2H]^{2+}$. **HR-MS** (ESI-TOF, +eV): m/z 1385.4547 (calculated for $[M+H]^{+}$); 1385.4545 (found). Purity by **HPLC** = 91% (Rt = 26.6 min).

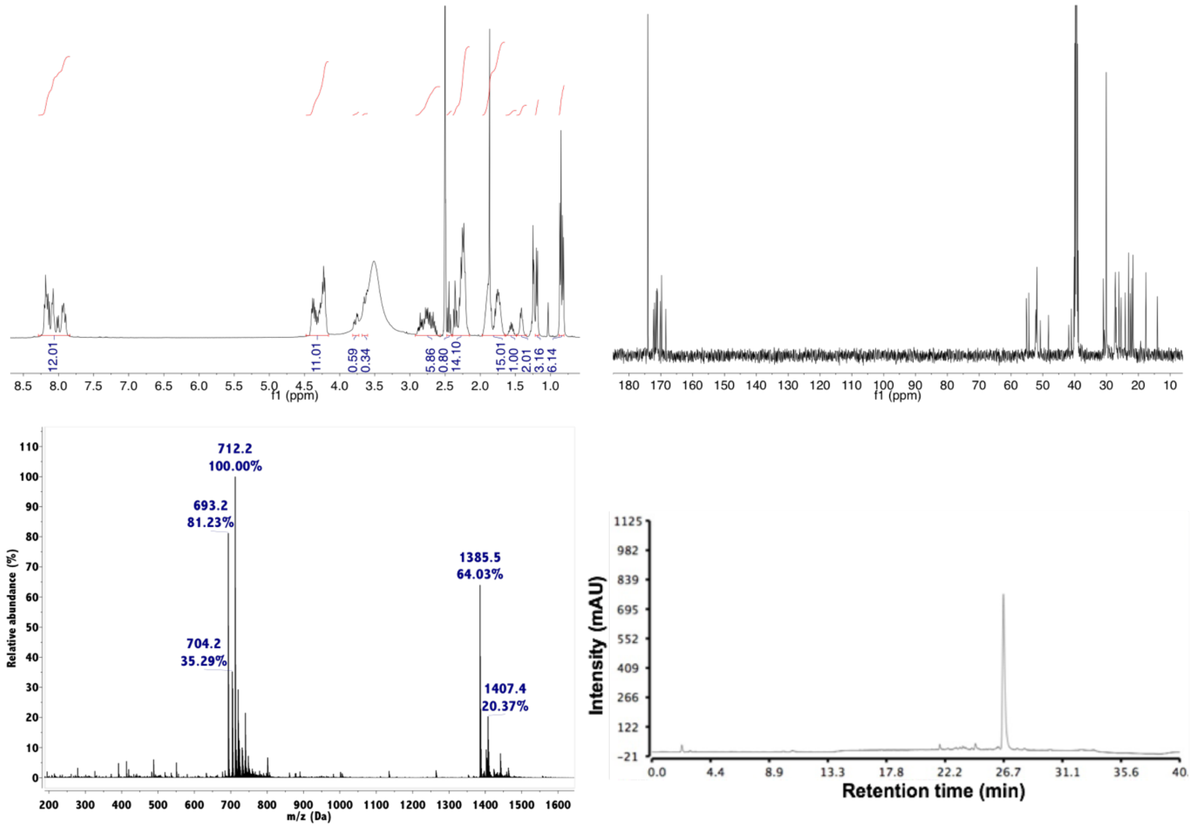

Figure S3 Characterisation of peptide **P4$_{SH}$** (Ac-C-EE-C-E-GLA-EEE-C-OH). Top: ^{1}H (left) and ^{13}C (right) NMR spectra (400 MHz, DMSO-*d$_6$*). Bottom: Mass spectrum (left) and RP-HPLC chromatogram (right).

5. Enzymatic degradation of peptides

In this experiment several controls have been performed to ensure that the observed increase in fluorescence is related to the elastase-mediated hydrolysis of the peptides.

- Succinyl casein (SC) is a positive control for enzymatic activity. This protein is degraded by most proteases, and an increase in fluorescence for SC in the presence of elastases indicates that these enzymes are active.
- All substrates (*e.g.* peptide, SC) have been incubated for 4 h under assay conditions in the absence of the elastases and the fluorescence observed is represented by black bars. This includes buffer alone (black bar now labelled control).
- **P5**, a peptide with a sequence that matches the hydrolysis product of one of our previously reported peptides,[4] was incubated in the absence of elastase and the fluorescence obtained was associated to 100% hydrolysis.

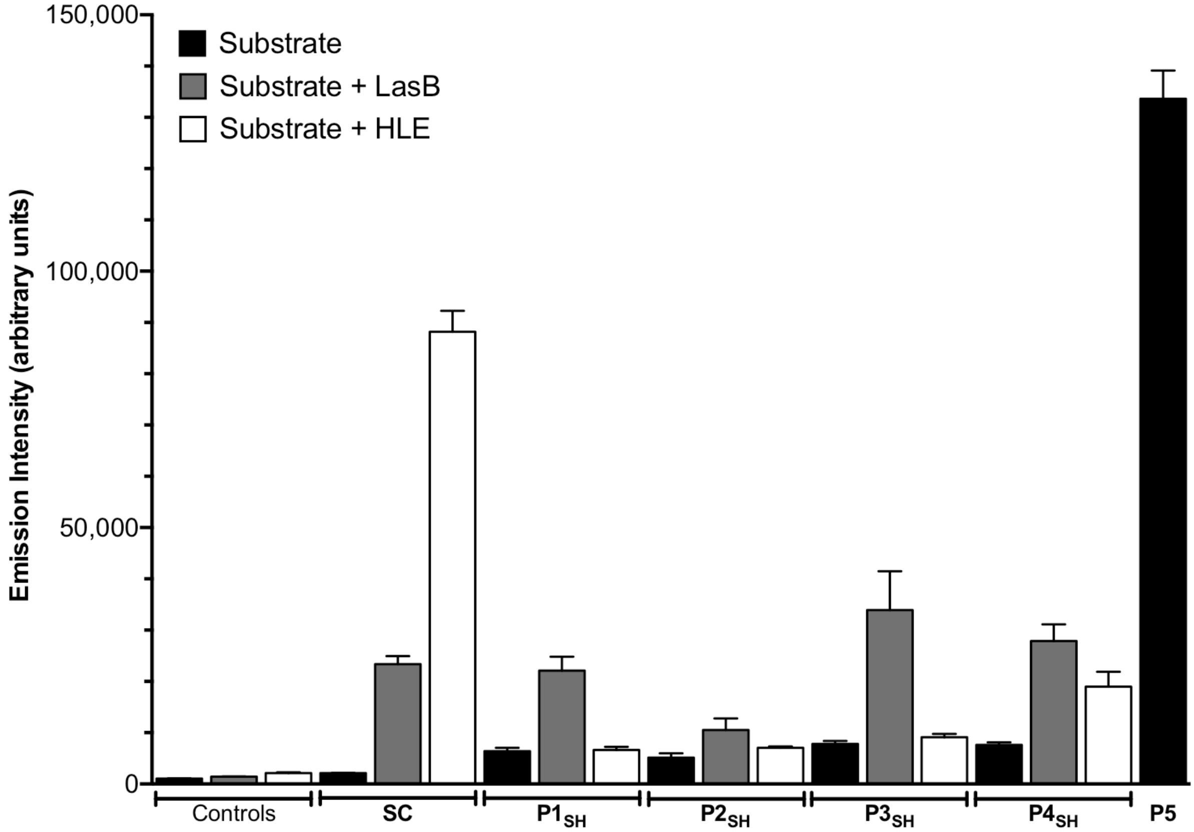

Figure S4 Emission intensity (λ_{exc} 355 nm, λ_{em} 460 nm) of fluorescamine conjugates for buffer and enzymes (controls), succinyl casein (SC, a control for enzymatic activity) in the absence and presence of enzymes, peptides **P1-4$_{SH}$** in the absence and presence of enzymes, and the peptide H_2N-LA-E-OH (**P5**, a control to normalise fluorescence intensity). All samples were incubated for 4 hours. *n* = 3, mean ± SD.

6. Circular dichroism characterisation of peptides

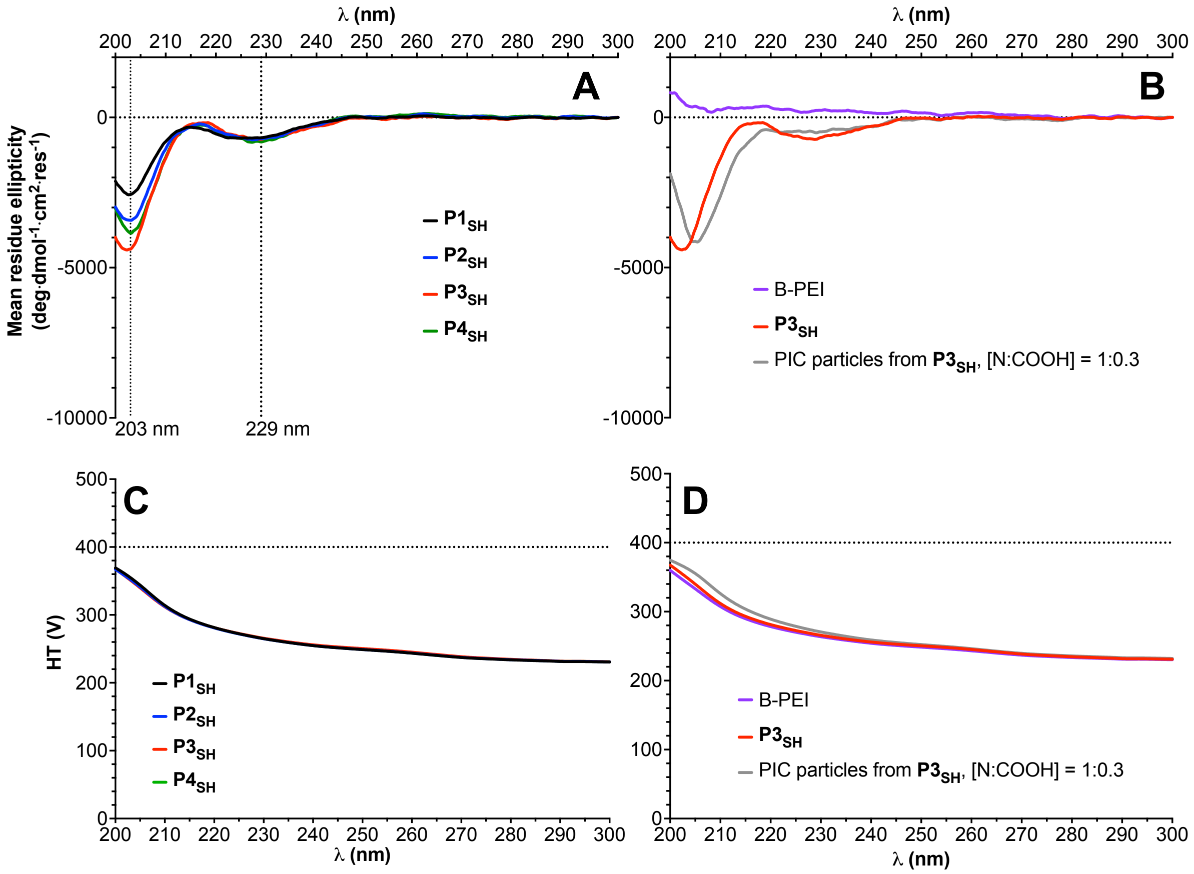

Figure S5 Top: Circular dichroism absorption spectra of peptides **P1**-**P4**$_{\mathbf{SH}}$ (A), and comparison between the spectra of **P3**$_{\mathbf{SH}}$, B-PEI and PIC particles prepared from these two materials at a 1:0.3 [N:COOH] ratio (B). Bottom: Dynode (HT) voltages recorded for the spectra above (C-D).

7. PIC particle characterisation

Table S1 Hydrodynamic diameter (D_H), polydispersity index (PDI) and ζ-potential of PIC particles prepared from peptides **P2-4$_{SH}$** and B-PEI at different [N:COOH] ratios. SD indicates the standard deviation found for the only size or charge population fitted by the software.

Peptide	[N:COOH] ratio	D_H ± SD (nm)	PDI [a]	ζ-potential ± SD (mV)	Notes
**P2$_{SH}$**	1 : 2.0	371 ± 51	0.02	-9.6 ± 3.9	-
	1 : 1.5	329 ± 42	0.02	-10.0 ± 3.1	-
	1 : 1.0	293 ± 30	0.01	-9.1 ± 3.5	-
	1 : 0.8	-	-	-	No particles found
	1 : 0.7	-	-	-	No particles found
	1 : 0.6	-	-	-	No particles found
	1 : 0.5	-	-	-	No particles found
	1 : 0.4	419 ± 67	0.03	+11.7 ± 5.2	-
	1 : 0.3	91 ± 32	0.12	+19.1 ± 6.2	-
	1 : 0.2	-	-	-	No particles found
**P3$_{SH}$**	1 : 2.0	227 ± 50	0.05	-21.3 ± 6.3	-
	1 : 1.5	204 ± 51	0.06	-18.5 ± 6.4	-
	1 : 1.0	198 ± 47	0.06	-18.3 ± 6.7	-
	1 : 0.8	212 ± 44	0.04	-16.5 ± 6.0	-
	1 : 0.7	224 ± 45	0.04	-16.0 ± 6.8	-
	1 : 0.6	280 ± 55	0.04	-15.8 ± 4.8	-
	1 : 0.5	-	-	-	No particles found
	1 : 0.4	240 ± 47	0.04	+13.9 ± 5.1	-
	1 : 0.3	103 ± 42	0.17	+21.3 ± 7.9	-
	1 : 0.2	-	-	-	No particles found
**P4$_{SH}$**	1 : 2.0	198 ± 45	0.05	-20.6 ± 6.1	-
	1 : 1.5	192 ± 43	0.05	-20.6 ± 7.2	-
	1 : 1.0	191 ± 41	0.05	-20.2 ± 5.8	-
	1 : 0.8	173 ± 36	0.04	-17.0 ± 7.4	-
	1 : 0.7	176 ± 39	0.05	-17.7 ± 7.2	-
	1 : 0.6	205 ± 39	0.04	-11.1 ± 7.5	-
	1 : 0.5	-	-	-	No particles found
	1 : 0.4	225 ± 46	0.04	+15.2 ± 5.4	-
	1 : 0.3	143 ± 40	0.08	+17.7 ± 7.7	-
	1 : 0.2	-	-	-	No particles found

The size and charge of PIC nanoparticle formulations prepared from peptide **P1$_{SH}$** has been previously reported.[3] [a] PDI values were calculated from the D_H and SD obtained by *General Purpose* (Malvern Instruments Ltd.) deconvolution of DLS correlograms using the following formula: PDI = $(SD/D_H)^2$.[5]

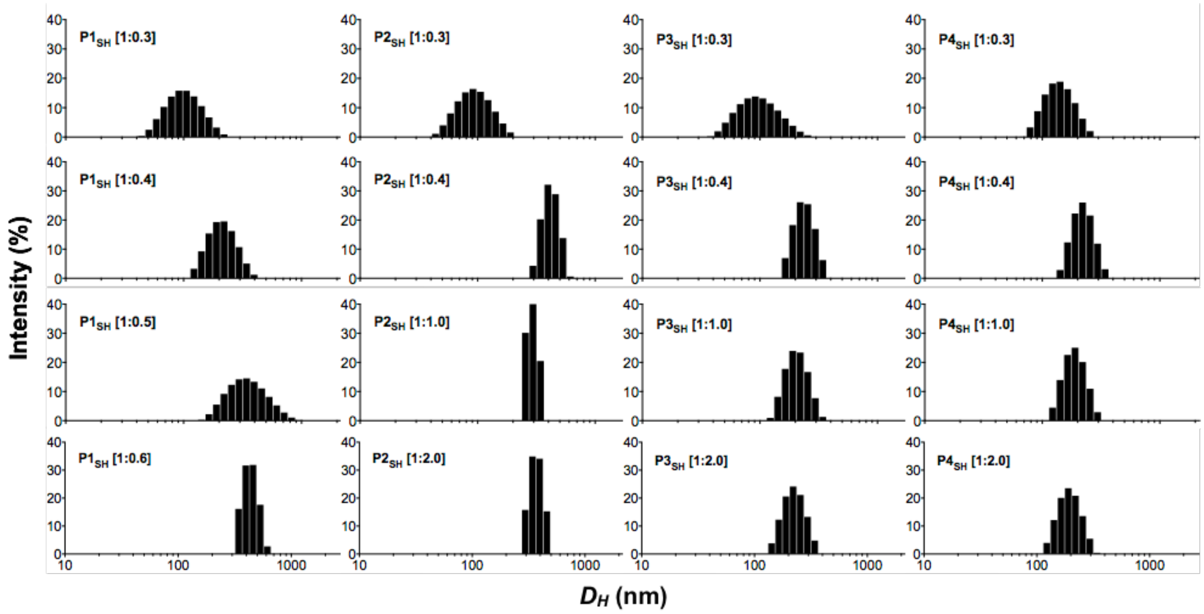

Figure S6 Representative DLS size-intensity distributions of freshly prepared PIC nanoparticles containing peptides **P1$_{SH}$-P4$_{SH}$** at different N:COOH ratios.

8. SLS characterisation of unfiltered PIC particles

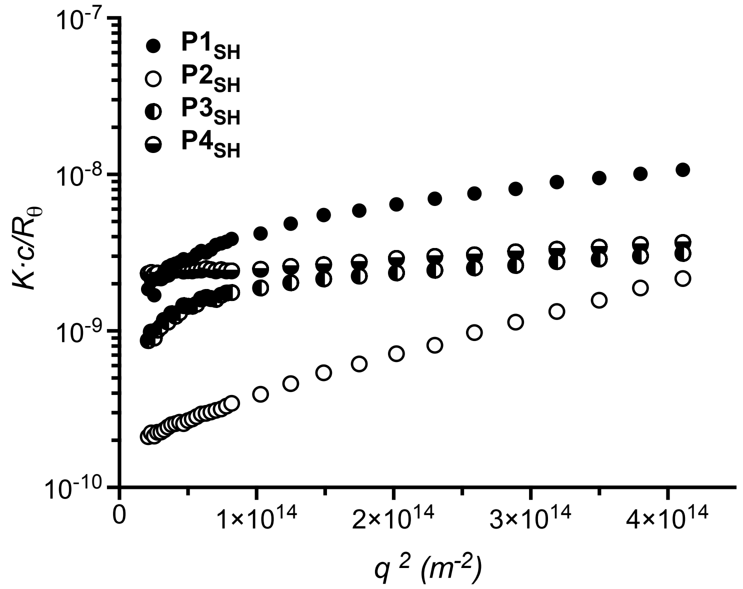

Figure S7 Partial Zimm plots obtained by SLS of unfiltered PIC particles prepared from peptides **P1-4$_{SH}$** at a 1:0.3 [N:COOH] ratio.

9. Shelf stability of PIC particles

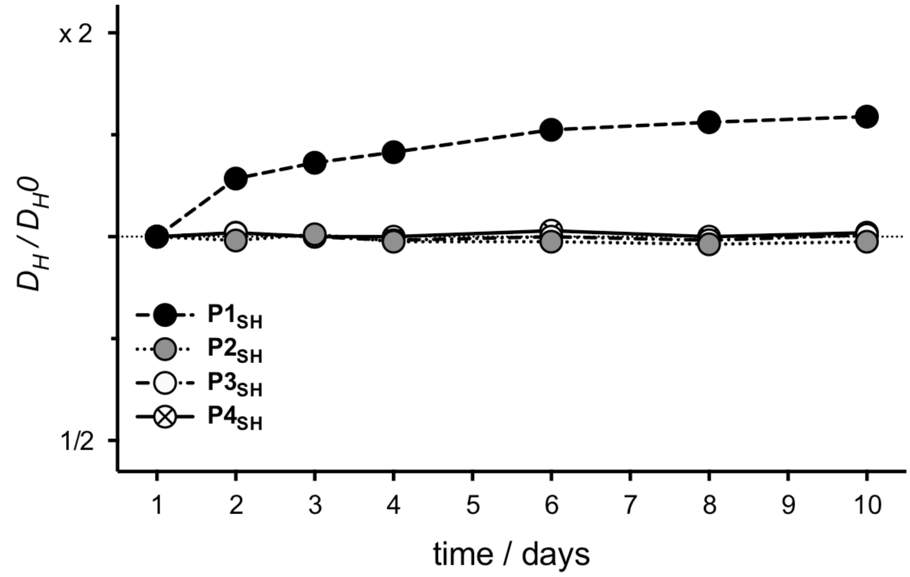

Figure S8 Relative change in size (*D_H / D_H0*) over time of PIC particles prepared at a 1:0.3 [N:COOH] ratio, stored in a dark, cool and dry place. The *D_H* values calculated by DLS were normalised to that of freshly made PIC particles (day 1).

10. Stability of the particles under physiological conditions

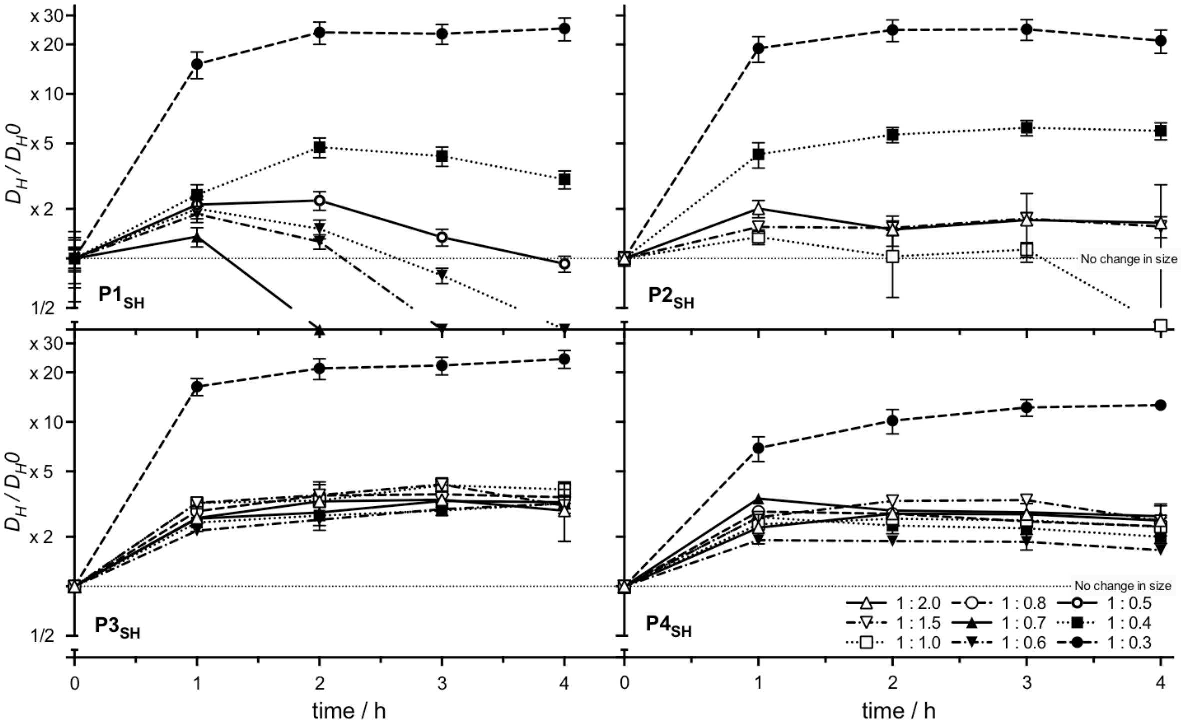

Figure S9 Relative change in size (*D_H / D_H0*) for PIC particles prepared from peptides **$P1_{SH}$-$P4_{SH}$** at representative N:COOH ratios under simulated physiological conditions (37 °C, 154 mM NaCl, pH 7.4)). Particle size (*D_H*) were normalised to that of the PIC particles in the absence of NaCl (0 hours, No change in size). *n* = 3, mean values ± standard deviation. Results obtained directly after the assembly of the nanoparticles without prior filtration. Data for **$P1_{SH}$** reproduced from I. Insua, E. Liamas, Z. Zhang, A. F. Peacock, A. M. Krachler, F. Fernandez-Trillo, *Polym Chem* **2016**, *7*, 2684–2690 - Published by The Royal Society of Chemistry.

11. Additional references

[1] N. J. Greenfield, *Nat. Protoc.* **2006**, *1*, 2876–2890.
[2] T. Vojkovsky, *Pept. Res.* **1995**, *8*, 236–237.
[3] I. Insua, E. Liamas, Z. Zhang, A. F. A. Peacock, A. M. Krachler, F. Fernández-Trillo, *Polym. Chem.* **2016**, *7*, 2684–2690.
[4] K. Morihara, H. Tsuzuki, *Arch. Biochem. Biophys.* **1971**, *146*, 291–296.
[5] Malvern Instruments Ltd., *Application of Dynamic Light Scattering (DLS) to Protein Therapeutic Formulations: Principles, Measurements and Analysis - 3. DLS Deconvolution Algorithms*, **2014**.
